# Guanylyl cyclase-B plays an important role in adipogenesis and “browning” of white fat

**DOI:** 10.1186/2050-6511-14-S1-P24

**Published:** 2013-08-29

**Authors:** Anja Glöde, Ana Kilić, John C Burnett, Alexander Pfeifer

**Affiliations:** 1 Institute of Pharmacology and Toxicology, Sigmund-Freud-Str. 25, 53105 Bonn, Germany; 2 Cardiorenal Research Laboratory, Mayo Clinic, Rochester, USA

## Background

Obesity, the excessive accumulation of adipose tissue, is the result of an imbalance in energy intake and output. Two functionally distinct types of adipose tissue are present in mammals: white adipose tissue (WAT) and brown adipose tissue (BAT). Beside its role as energy storage, WAT is also a metabolic active organ involved in inflammatory response, glucose metabolism and blood pressure by secreting adipokines. In contrast to WAT, BAT dissipates energy as heat. Previously, we have demonstrated essential role of the cGMP/PKGI in brown and white adipogenesis.

## Results

In the present study, we investigated pathways upstream from cGMP/PKGI, namely natriuretic peptides (NPs) and their receptors in adipogenesis. All three receptors for NPs (GC-A, GC-B and the clearance receptor NPR-C) are expressed in murine BAT and WAT as demonstrated by real-time PCR. Interestingly, levels of GC-A were significantly lower than levels of GC-B and NPR-C. Stimulation of brown adipocytes with CNP resulted in a significantly enhanced adipogenesis as shown by increased Oil RedO staining of accumulated intracellular lipids and measurement of triglyceride (TG) content. In addition, expression of adipogenic marker proteins (PPARy and aP2) as well as thermogenic marker protein (UCP-1) was significantly increased after 200 nM CNP treatment (by 1.9 fold, 1.8 fold, 1.7 fold respectively). Novel designer natriuretic peptide CD-NP, a hybrid of C-type natriuretic peptide (CNP) and *Dendroapsis* NP (DNP), proved as the most potent natriuretic peptide, since 100 nM of CD-NP caused the highest increase in adipogenesis and thermogenesis of brown adipocytes as measured by western blot analysis (2.8 fold for PPARy, 3.3 fold for aP2 and 2.8 fold for UCP1). Taken together CD-NP exerts positive adipogenic effect on primary white adipocytes (WA). Interestingly, CD-NP caused enhanced “browning”, i.e. increased UCP-1 expression in WA by (2.06 fold).

## Conclusion

In conclusion, CNP and novel designer natriuretic peptide CD-NP promote adipogenesis and thermogenesis in brown and white adipocytes. Due to the positive effects on adipogenesis and thermogenesis, CD-NP could be considered as a novel drug for treatment of obesity.

